# Automated generation of gene summaries at the Alliance of Genome Resources

**DOI:** 10.1093/database/baaa037

**Published:** 2020-06-19

**Authors:** Ranjana Kishore, Valerio Arnaboldi, Ceri E Van Slyke, Juancarlos Chan, Robert S Nash, Jose M Urbano, Mary E Dolan, Stacia R Engel, Mary Shimoyama, Paul W Sternberg, the Alliance of Genome Resources

**Affiliations:** 1 WormBase, Division of Biology and Biological Engineering, California Institute of Technology, 1200 East California Boulevard, Pasadena, CA 91125, USA; 2 ZFIN, The Institute of Neuroscience, 222 Huestis Hall, University of Oregon, Eugene, OR 97403-1254, USA; 3 *Saccharomyces* Genome Database, Department of Genetics, Stanford University, 3165 Porter Drive, Palo Alto, CA 94304, USA; 4 MGI, The Jackson Laboratory, Bar Harbor, ME 04609, USA; 5 FlyBase, Department of Physiology, Development and Neuroscience, 7 Downing Pl, University of Cambridge, Cambridge CB2 3DY, UK; 6 Rat Genome Database, Department of Biomedical Engineering, Medical College of Wisconsin and Marquette University, 8701 Watertown Plank Road, Milwaukee, WI 53226, USA

## Abstract

Short paragraphs that describe gene function, referred to as gene summaries, are valued by users of biological knowledgebases for the ease with which they convey key aspects of gene function. Manual curation of gene summaries, while desirable, is difficult for knowledgebases to sustain. We developed an algorithm that uses curated, structured gene data at the Alliance of Genome Resources (Alliance; www.alliancegenome.org) to automatically generate gene summaries that simulate natural language. The gene data used for this purpose include curated associations (annotations) to ontology terms from the Gene Ontology, Disease Ontology, model organism knowledgebase (MOK)-specific anatomy ontologies and Alliance orthology data. The method uses sentence templates for each data category included in the gene summary in order to build a natural language sentence from the list of terms associated with each gene. To improve readability of the summaries when numerous gene annotations are present, we developed a new algorithm that traverses ontology graphs in order to group terms by their common ancestors. The algorithm optimizes the coverage of the initial set of terms and limits the length of the final summary, using measures of information content of each ontology term as a criterion for inclusion in the summary. The automated gene summaries are generated with each Alliance release, ensuring that they reflect current data at the Alliance. Our method effectively leverages category-specific curation efforts of the Alliance member databases to create modular, structured and standardized gene summaries for seven member species of the Alliance. These automatically generated gene summaries make cross-species gene function comparisons tenable and increase discoverability of potential models of human disease. In addition to being displayed on Alliance gene pages, these summaries are also included on several MOK gene pages.

## Introduction

Often, a key task for biological and biomedical knowledgebases is the summarization of the knowledge about a gene. Gene summaries serve as a quick introduction to gene function, providing a high-level picture of the gene and its biological role. A textual gene summary is user-friendly, requiring no knowledge of controlled vocabularies such as ontologies, or of knowledgebase-specific data models. Summarizing the knowledge about a gene is usually done by writing a brief text summary that condenses and arranges all of the current knowledge about that gene into several data categories. These categories often include molecular function (MF) or activity, biological processes (BPs) in which the gene is involved, orthology/homology data and gene expression at the tissue and subcellular levels. Curators may add additional gene information such as pathway data, genetic and physical interactions, drug interactions and regulation of gene expression and activity.

Several model organism knowledgebases (MOKs) such as *Saccharomyces* Genome Database (SGD; 1) and WormBase (WB; 2) have manually written gene summaries (3, 4), while some, such as FlyBase (FB; 5), solicit them from their communities (6). The manual writing of gene summaries is time-consuming and labor-intensive. In addition, it is difficult for curators to update existing summaries as new data become available. To address this problem, MOKs such as WB and Rat Genome Database (RGD; 7) moved to an automated method (1, 7). WB generated gene summaries by applying sentence templates on highly structured data such as gene annotations to the Gene Ontology (GO; 8), the Disease Ontology (DO; 9) and WB anatomy ontologies (AOs) and applied a simple cutoff strategy to handle genes with long lists of annotated terms. In these cases, only a few terms randomly chosen by the algorithm would appear in the final summary, resulting in a loss of information that was not apparent to the reader (10). Similar to WB, RGD used a template-based method to automatically generate gene summaries that prioritized terms based on evidence codes defined by the Evidence and Conclusion Ontology (ECO; 11), with a maximum of three terms per data category. When more than three terms were present, three would be chosen randomly. Gene summaries were generated on demand when a gene report page was loaded and weekly when the gene FTP files were produced (7). Mouse Genome Database (MGD; 12) had produced detailed auto-generated gene summaries for a number of years, based on the complete set of their GO annotations, without limiting the number of terms displayed in the summaries. However, as the volume of GO data grew, many of the summaries became too long to be of practical use as an overview of gene function (13).

Other groups have generated gene summaries from the biomedical literature by first identifying and retrieving the relevant articles for specific genes and then extracting the most representative sentences for the specified semantic categories that describe the gene (14, 15). Compared with these methods, automated gene summary generation from curated data has the advantage of being based on key gene information selected by either human curators or specialized software. In addition, by using standard ontologies, gene-related annotations can be generalized by traversing the ontologies and grouping terms by their common ancestors. This allows summaries to reach the right balance between coverage of all the annotations to a gene and granularity of the provided information. On the other hand, in the methods described above (14, 15), sentences are extracted from pre-existing text in the literature and simply prioritized based on their content. However, related information in the sentences is not combined.

The Alliance of Genome Resources (www.alliancegenome.org; Alliance; 16) is a portal for common data curated by the founding members: the GO and six MOKs [MGD, RGD, Zebrafish Information Network (ZFIN; 17), FB, WB and SGD]. In order to generate automated, standardized and modular text summaries for all genes of member species of the Alliance and to overcome the limitations of existing gene summary software, we developed a new method for automatically generating gene summaries. Similar to previously described gene summary generation methods (1, 7, 10, 12), the approach described in this paper is based on an algorithm that uses sentence templates that include verb phrases (e.g. ‘involved in’, ‘exhibits’) followed by the list of terms annotated to the gene of interest. The key feature of this method is its ability to ‘trim’ long lists of ontology terms annotated to a gene, by grouping terms via their common ancestors drawn from the source ontology. The trimming algorithm selects the best combination of the initial set of terms, or their ancestors, to be included in the final gene summary. This is done via an optimization process that balances readability of a gene summary with the amount of information it provides. We designed two different trimming algorithms. One chooses the best combination of lowest common ancestors (LCAs) (18) of the set of ontology terms annotated to the gene that provide the highest coverage of the initial terms. The second algorithm chooses the best combination of ancestor terms (not limited to LCA), based not only on their coverage but also on measures of their information content (IC). We describe these algorithms and provide a comparative analysis to assess their performances.

The automated gene summaries generated by our method include gene functional data for seven species, six MOK species, in addition to human data provided by RGD, which maintains a full set of human gene records and annotations imported and integrated from diverse sources such as NCBI (19), Ensembl (20), UniProt-GOA (21) annotations mapped to HGNC IDs (HUGO Gene Nomenclature Committee; 22) and Online Mendelian Inheritance in Man (23).

The automated gene summaries are based on highly structured gene data such as annotations to ontology terms from several different ontologies [e.g. Gene Ontology, http://geneontology.org/ (8); Disease Ontology, http://disease-ontology.org/ (9); see Materials and Methods below for the full list of ontologies and data categories included in the summaries]. The gene summaries highlight gene MFs, BPs, expression data and gene relevance to human health and disease in a succinct readable form. The summaries benefit MOK users by enabling cross-species comparison, and they help clinical researchers and human geneticists who are unfamiliar with specialized MOK vocabularies by aiding in the discoverability of potential models of human disease.

The gene summaries pipeline described in this paper has been in production at the Alliance since March 2018. Over 121 000 gene summaries are available for the seven species in the 2.3 version of the Alliance release. They can be downloaded in bulk from the Downloads page on the Alliance website (https://alliancegenome.org/downloads#gene-descriptions), and they are displayed on current Alliance gene pages. In addition, Alliance summaries are currently imported for display on MOK gene pages that had no gene summaries in the past (ZFIN), supplement existing summaries at the other MOKs (MGD, WB, FB and RGD), and are used to enhance existing curated summaries (SGD).

## Materials and Methods

### Data categories in gene summaries and source of annotations

We generated automated gene summaries using gene annotations to ontology terms such as GO, DO, etc. and additional gene-related data such as orthologs. For a list of data categories in the gene summaries and the annotations/ontologies they are based on, see Table 1. Note that all data used in the gene summaries are obtained from the Alliance File Management System API (https://fms.alliancegenome.org) and from the Alliance database at build ime.

**Table 1:** Data categories used to generate gene summaries and their sources

Data category in gene summary	Source of ontology/annotations
Molecular function	GO molecular function ontology (8)
Biological process	GO biological process ontology (8)
Subcellular localization	GO cellular component ontology (8)
Disease relevance	Disease Ontology (9)
Disease biomarker	Disease Ontology (9)
Human ortholog implicated in disease	Alliance orthology data (16) and Disease Ontology (9)
Tissue/cellular expression	MOD-specific anatomy ontologies—Zebrafish Anatomy and Development Ontology (24), WB Anatomy Ontology (25), Drosophila Anatomy ontology (26), Mouse Developmental Anatomy Ontology (27)
Orthology to human gene	Alliance strict orthology set (15)

### Selecting ontology-based annotations

#### Selecting annotations

All gene annotations to ontology terms that MOKs submit to the Alliance for a gene of interest were considered for generating gene summaries. However, in order to maintain readability of the final description, when a gene had annotations supported by experimental evidences (according to ECO; 11), these annotations were preferred over others. When a gene had no experimental annotations, annotations supported by other types of evidences were included such as the following (in order of preference): high-throughput experimental evidence codes, phylogenetic evidence codes, curator and author statement evidence codes, computational evidence codes and electronic evidence codes (8, 11, 28).

#### Excluding annotations

Some ontology terms exist in an ontology in order to make the ontology complete, and these terms are clearly not suitable for inclusion in a gene summary. We manually ‘blocklist’ such terms (if found annotated to a gene) and remove them from the list of terms used for the summaries.

In addition, developers of ontologies maintain lists of terms that are not suitable for use in the annotation of a gene but can be used for grouping terms. For example, the GO consortium maintains a list of terms to which annotations should not be made (i.e. ‘do not annotate’ and ‘do not manually annotate’ lists on their website: http://geneontology.org/docs/download-ontology/). We exclude direct annotations to these terms from the gene summaries, but we do not add them to the blocklist, allowing the algorithm to choose them as grouping terms while trimming, as explained in more detail below (see section on ‘Trimming summaries for readability’).

### Selecting orthologs

We include only human orthologs in the gene summary. Human orthologs come from the ‘stringent ortholog set’ computed at the Alliance that is a set of orthologs based on the integration of different orthology prediction methods by the DRSC Integrative Ortholog Prediction Tool (15, 29). From the Alliance stringent ortholog set, we select only those orthologs that have the highest number of prediction methods (i.e. the ‘best’ orthologs) for inclusion in a gene summary, up to three. When more than three best orthologs exist, we show the first three and exclude the others. For each ortholog in a gene summary, we include both its symbol and name.

### Sentence templates for data category specific summaries

Templates were built for the generation of sentences for each data category by using the actual ontology terms that the gene of interest is annotated to, together with a verb phrase such as ‘exhibits’, ‘involved in’, ‘is expressed in’, etc. To describe the orthology of a MOK gene to human orthologs, we use the phrase ‘Orthologous to human’ followed by the list of orthologs. For the complete list of sentence templates for each data category and the maximum number of ontology terms used in the summary, see Table 2. When more than two ontology terms or orthologs are included in the summaries, they are separated by semicolons because many of their names contain commas (e.g. the GO term ‘non-replicative transposition, DNA-mediated’). For the sake of readability, a maximum of three terms were included for each data category except for the categories of expression and disease, where five terms were included in the final summary based on ontology structure and curator evaluation.

**Table 2:** Sentence templates for data categories

Data category	Sentence template
Molecular function	Exhibits <list of GO MF terms>
Biological process	Involved in <list of GO BP terms>
Subcellular localization	Localizes to <list of GO CC terms>Localizes to <list of GO CC terms>
Disease relevance	Used to study <list of DO terms> Biomarker for <list of DO terms>
Implication of human orthologs (of MOK gene of interest) in disease	Human ortholog(s) of this gene implicated in <list of DO terms>
Expression	Expressed in <list of AO terms>
Human orthologs	Orthologous to <list of human orthologs>

Sentence templates that contain a verb phrase and the ontology terms annotated to the gene of interest were used to generate data category-specific summaries. The data categories of molecular function, biological processes a gene is involved in and sub-cellular localization are based on terms annotated to the gene from the GO ontologies of MF, BP and cellular component (CC). Human disease relevant data are based on gene and allele annotations to terms from the DO and gene expression data are based on gene annotations to terms from the MOK specific AOs. Orthology data is based on the stringent ortholog set computed at the Alliance and an additional filter that takes into account the number of ortholog prediction methods that have selected the ortholog.

**Figure 1:**
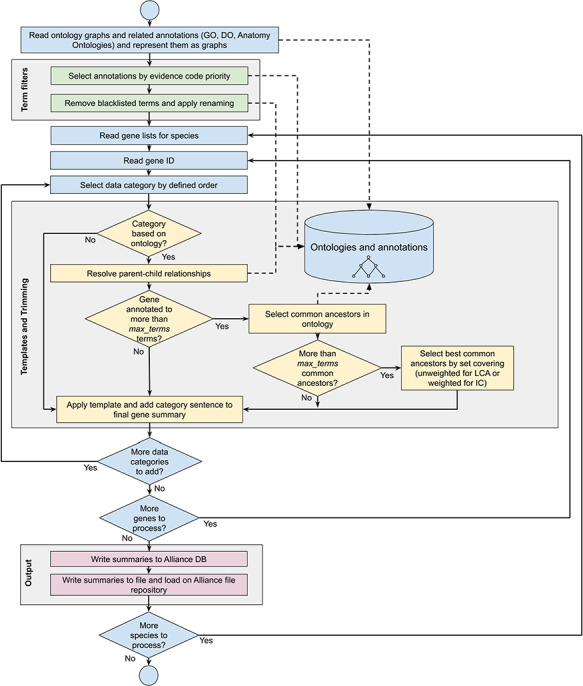
Workflow diagram of the gene summary generation process at the Alliance of Genome Resources. Solid arrows represent sequential steps followed by the software to generate the summaries, whereas dashed arrows represent data flow from/to the algorithm to data stores. Ontologies and annotations are loaded and represented as graphs. Then, term filters and renaming are applied to the annotations and to the ontology graphs. For each gene, basic information such as gene ID, name and additional information such as orthology data are fetched. The list of terms associated with the gene is extracted from the Alliance database and sentences are generated according to the templates defined for each data category. If the list of terms exceeds the defined maximum number, the trimming algorithm reduces the length of the sentence by traversing the related ontology graph and by selecting the common ancestors that best group the initial set of terms. Ontology graphs are also used to resolve parent–child relationships to avoid including both parent and child terms in the final summaries. The final summaries are generated by concatenating the data category specific sentences and are written to the Alliance database and to the download files available on the Alliance website.

When a gene is annotated to numerous ontology terms that exceed the maximum number for a given data category, the number of terms is reduced by applying a trimming algorithm (see below section ‘Trimming summaries for readability’). If the number of terms after trimming still exceeds the maximum number, only the best combination of terms is included based on an optimization algorithm (also described below) and the other terms are excluded. The trimming algorithm selects ancestor terms in the ontology to cover more specific terms in the initial annotation set, balancing readability of the summaries with the amount of information they provide. As shown in detail in the section ‘Trimming summaries for readability’, a high coverage of the initial set of annotations is obtained with a sufficiently high level of granularity of the terms in the final summaries, meaning that the loss of information caused by the trimming algorithm is marginal. For summaries where trimming is applied, an alternative sentence template is used for each of the data categories that indicate to the reader that not all of the data are included in the summary. For example, for the MF summary the following template is used:

Exhibits several functions, including <ontology terms selected>.

Similarly, when the number of best orthologs exceeds the maximum number of orthologs to be included in the summary, we apply the following templates (and other variations of the template for data categories not listed here):

Orthologous to several human genes, including <list of orthologs>,

Expressed in several structures, including <list of AO terms>.

For annotations based on non-experimental evidence codes, such as those derived from phylogenetic, sequence based or computational analyses [e.g. GO annotations that are a result of projects such as PAINT and INTERPRO2GO (28, 30, 31)] the phrase ‘Predicted to’ is prepended to the verb phrase of the template, e.g.

Predicted to exhibit <list of GO molecular function terms>.

For molecular function, BP and cellular component annotations, the sentence template is modified if a qualifier (31) is present:

Contributes to <list of terms> (for contributes_to GO qualifier),

Colocalizes with (for colocalizes_with GO qualifier).

For the list of sentence templates for each data category, see Table 2. All criteria such as the priority of type of annotations, number of ontology terms to include in the final summary and sentence templates for each data category were defined based on discussions with data specific curators at the Alliance.

### Building the final gene summary

A gene summary is built for a given gene of interest using the (i) pre-defined rules for selecting annotations as described previously, (ii) pre-defined sentence templates for each data category and (iii) trimming the number of terms in a given data category when the number of terms exceeds the predefined number. See Figure 1 for the gene summary generation workflow implemented at the Alliance.

**Figure 2:**
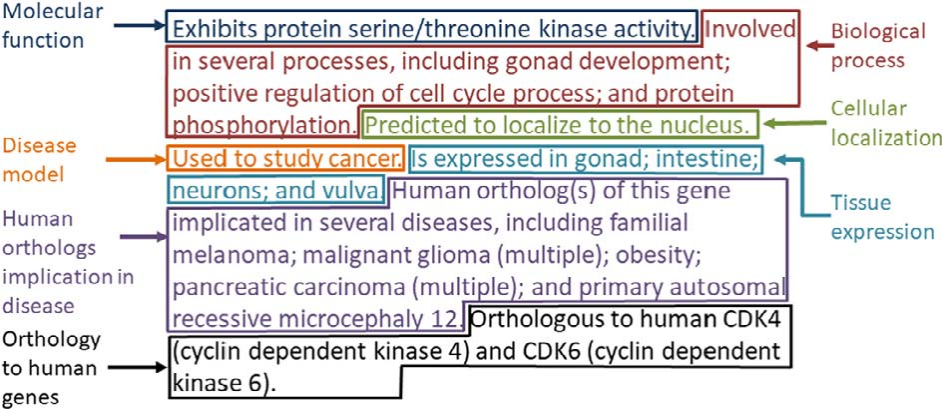
Example of the gene summary for the *C. elegans* gene *cdk-4* with the different data categories highlighted in different boxes.

The gene summary obtained by the concatenation of the individual data category specific summaries is shown for the *Caenorhabditis elegans* (C. elegans) gene *cdk-4* in Figure 2. Note that, even though each data category can only have either experimental or predicted data due to the evidence code prioritization, the final summary may contain a mixture of experimental and predicted annotations across different categories, as is the case for the *C. elegans* gene *cdk-4*, shown in Figure 2.

### Trimming summaries for readability

When a gene is annotated to more than the maximum number of terms allowed for a given data category, we trim the initial list of terms annotated to a gene to a specified maximum number that can be set for each data category in the summary. Before trimming, when both parent and child terms are present in the initial annotation set, one of them is removed based on the data category. For data categories based on GO, the parent GO term(s) are removed in order to keep the most granular term. For tissue expression, the child terms are removed if a parent is present, in order to avoid long lists of cells or tissue parts in the final gene summary. Trimming takes advantage of the design of biomedical ontology graphs where ancestor terms represent more generic concepts than the granular terms below them.

For a given initial set of ontology terms there can be one or more common ancestors, including the root term. However, the root term and many of its high-level descendents are not informative enough to be included in the final gene summaries, e.g. the root term biological process in the GO BP ontology. Even ‘slims’, provided by ontology developers such as GO (http://geneontology.org/docs/go-subset-guide/), contain terms that are high level and not informative enough for the summaries. For these reasons, we decided to select only the ancestor terms at a predetermined minimum distance from the root term. This minimum distance was determined based on a manual inspection of each of the ontologies. We understand that using a fixed minimum distance from the root does not take into account the granularities that different ontology branches can have. For this reason, one of the trimming algorithms that we developed (introduced below and used in production at the Alliance) uses measures of IC of the terms to select grouping terms at the optimal distance from the root. Nonetheless, the predetermined minimum distance from the root is used in cases where IC is not sufficient to avoid high-level terms, for example when the number of annotations to a gene is very high (e.g. the *C. elegans* gene *daf-16*).

Since there can be multiple paths from a term in the ontology to the root, the distance between terms is calculated as the length of the longest path connecting them. Only ‘is_a’ and ‘part_of’ relationships (defined in the Relation Ontology; https://github.com/oborel/obo-relations) in the ontologies were considered for the selection of common ancestors (http://geneontology.org/docs/ontology-relations/).

For the disease data category, common ancestor terms that cover more than one initial term are followed by the word ‘multiple’ in parentheses when they are included in the final summary (Figure 2). This is to indicate to the reader that the gene is associated with several specific types of the disease indicated in the summary (e.g. ‘pancreatic cancer’ covers ‘pancreatic adenocarcinoma’, ‘pancreatic ductal adenocarcinoma’ and other types of pancreatic carcinomas).

We developed two different types of trimming algorithms: (i) trimming by choosing the LCA and (ii) trimming by choosing ancestor terms weighted by IC.

### Trimming by choosing LCAs

Given an initial set of terms, this trimming algorithm returns the LCAs of the terms (18) to be included in the final summary.

**Figure 3:**
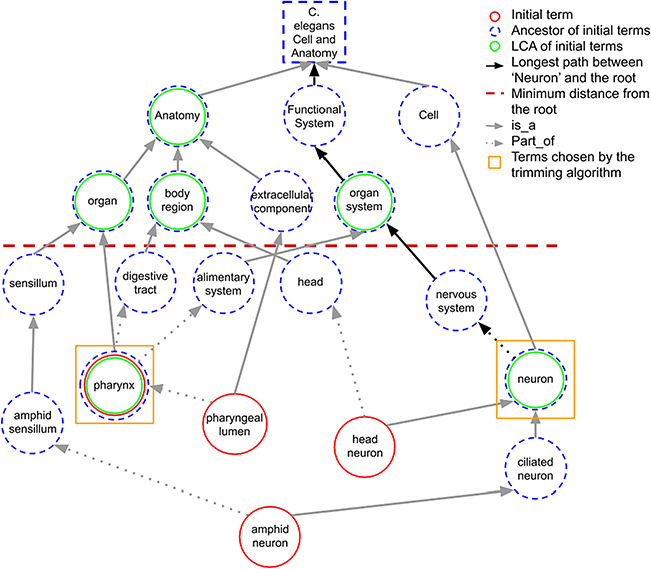
A portion of the *C. elegans* anatomy ontology graph (generated with the WB SObA tool; 33). Terms circled in red (single solid circle) represent the initial set of annotated terms for the *C. elegans* gene *abf-1*. Dashed blue circles are the ancestors of the initial terms and green circles (double circle) are their respective LCAs in the ontology (excluding the root node). The terms pharynx and neuron (marked by yellow squares) are chosen by the trimming algorithm as they are the only LCAs at the predefined minimum distance from the root (depicted as a dashed horizontal line), which in this example is set to 3. Note that the distance of a term from the root is the length of the longest path between them, which is highlighted in the figure as an example for the term neuron.

When a term in the initial set does not share any ancestors in common with other initial terms at the predefined minimum distance, it is considered as the LCA of itself. Figure 3 shows how LCA terms are identified from a set of initial terms, given a predefined minimum distance from the root term set to 3. The figure shows how the best terms are selected for tissue expression from the *C. elegans* AO for gene *abl-1*.

If the number of LCAs is not greater than the predetermined maximum number of terms (*max_terms*), all the LCAs are included in the final summary. Otherwise, the trimming algorithm selects the best subset of LCAs through an optimization step. The selection of the smallest possible combination of ancestors that covers the initial set of terms (i.e. which are connected, directly or indirectly, to the terms) is formulated as a set covering problem. This problem is then solved by a greedy algorithm well known in computer science (32). The algorithm iteratively selects the ancestor with the highest coverage of the initial set of terms, excluding the terms already covered in previous iterations. The algorithm can be halted when the predetermined max_terms is reached. However, in this case, full coverage of the initial terms may not be reached for every gene. To highlight this, as already explained above, the word ‘several’ is added to the template, indicating to the reader that some of the data may not be included in the summary (see Sentence templates section).

### Trimming by choosing ancestor terms weighted by IC

The set covering optimization algorithm can also work with ‘weighted’ terms. In this case, at each iteration of the algorithm, the measure of coverage of a term is multiplied by its weight in order to select the best combination of terms for the final gene summary. The weight of an ontology term is usually expressed in terms of its IC (34), defined as follows:

$$ IC(t)=-\mathit{\log}\left[p(t)\right], $$

where *t* is an ontology term and *p(t)* is the probability of finding *t* in a specific context (e.g. the term `head' in the context of biology). Several IC measures exist in the literature, each of which estimates *p(t)* differently (see IC measures section).

We designed a trimming algorithm that uses a weighted version of the set covering optimization based on IC. Unlike the trimming based on LCA, trimming based on IC considers all the possible common ancestors of the initial terms (not only the LCAs) and selects the best combination of ancestors and/or initial terms to be included in the final summary through the weighted optimization algorithm.

### IC measures

Several measures of IC have been proposed in the literature. The classic measure of IC (35) estimates *p(t)* as the frequency of appearance of a term (or its synonyms) in a large corpus such as a specific literature corpus, e.g. the *C. elegans* literature. This measure is independent of the structure of the ontology that the term belongs to, as well as the annotations of genes to the term, and is defined as follows:

$$ I{C}_{corpus}(t)=-\mathit{\log}\left(\frac{m(t)+1}{\sum_{t_i\in T}\left[m\left({t}_i\right)\right]+1}\right), $$

where *m(t)* is the number of times the term *t* or its synonyms appear in the corpus.

However, this measure is computationally expensive because the frequency of appearance of the term needs to be calculated on a corpus that usually contains a large number of documents. Even though the frequencies can be pre-calculated and stored in a file before running the gene summary software, the values may need to be recalculated to reflect any changes in the corpus, making this measure inflexible (36).

Some measures of IC have been proposed in the literature to overcome these limitations, including the two that we describe below and that we implemented in our software—*IC_annot_* and *IC_sanchez_*—which, to the best of our knowledge, provide the best results.

### IC measure based on number of annotations to a term (*IC_annot_*)

This IC measure (36) estimates *p(t)* as the fraction of genes annotated to *t* or to its descendants with respect to the total number of genes annotated to terms in the ontology:

$$ I{C}_{annot}(t)\!=\!-\textit{log}\left(\frac{\mid\! annot(t)\cup{\cup}_{di\in desc(t)}\left[ annot\left({d}_i\right)\!\right]\mid \!+\!1}{ \mid{\cup}_{z\in T}\left[ annot(z)\right]\mid +1}\!\right)\!, $$

where *annot(t)* is the set of genes annotated to *t* and *desc(t)* is the set of descendants of *t* in the ontology *T* [*annot(d_i_)* is the set of genes annotated to $d_i\in desc(t)$, one of the descendants of *t*]. This measure is easy to calculate and does not depend on the structure of the ontology, which may be incomplete. On the other hand, gene annotations to terms may be incomplete or biased toward specific areas of biology, depending on curation practices (36). In addition, more specific terms could be added to the ontology subsequent to the process of annotation, and therefore some of these new terms may have fewer annotations than they should have had, resulting in a biased IC value. Moreover, since each organism has a different set of annotations, the same ontology term may have different IC values for different organisms for different organisms.

### IC measure based on ontology structure (*IC_Sanchez_*)

This IC measure (34) is based on the structure of ontologies and estimates *p(t)* as a function of the structural properties of the ontology graph. It is defined as follows:

$$ I{C}_{sanchez}(t)=-\mathit{\log}\left(\frac{\frac{\left| leaves(t)\right|}{\left|A(t)\right|}+1}{maxleaves+1}\right)\!, $$

where $leaves(t)=\Big\{l\in T\ |\ l\in desc(t)\wedge l\ is\ a\ leaf\Big\}$, *l* is a leaf iff $desc(l)=\varnothing$ and *maxleaves* is the total number of leaves in the ontology.

This measure uses knowledge embedded in the ontology structure and overcomes limitations of IC based on annotations. However, it may be biased if the ontology is not fully and equally developed across different branches (areas of biology) (37).

### Gene summaries software package

The software for generating gene summaries is organized into a Python package available at https://github.com/alliance-genome/agr_genedescriptions and released under the MIT open source license (https://github.com/alliance-genome/agr_genedescriptions/blob/master/LICENSE.txt). The package contains functions to load data files and ontologies, generate summaries from the data, calculate statistics on the summaries and write both summaries and statistics to files. It is based on ontobio (https://github.com/biolink/ontobio), a flexible and multi-format library for managing ontologies and annotations. The gene summaries software is used at the Alliance and is integrated into the build process of the web portal to generate a summary on each gene page, and species-specific summary files for download in different file formats, including the custom gene summary JSON format defined at the Alliance (https://github.com/alliance-genome/agr_schemas/tree/master/ingest/genedescription). The package can also be used as an external dependency by custom pipelines, as done at WB (7).

A simple React JavaScript report tool was also developed for curators. The tool allows the display, download and comparison of gene summaries from different Alliance releases for the purposes of easily viewing and manually inspecting the summaries (available at https://github.com/alliance-genome/agr_genedescriptions_reporttool).

## Results

### Comparative analysis of different trimming algorithms

We compared the gene summaries obtained by the different trimming algorithms (unweighted version based on LCA and weighted version based on *IC_sanchez_* and *IC_annot_* using Alliance annotations), using data from Alliance v.2.3.0. In Table 3, we show the results of this comparison in terms of average percentage of coverage of the initial set of terms, in the final summaries. We also report the specificity of the final terms as a measure of both their depth in the ontology and their *IC_corpus_* value (based on the frequency of appearance of a term in a large corpus as defined in the IC measures section). The corpus we used to calculate *IC_corpus_* was the set of open access MOK papers (roughly 77 000 articles that represents the total set of papers collected by each MOK) downloaded from PubMed on 15 October 2019.

**Table 3:** Comparison between trimming algorithms

Species	Trimming algorithm	Avg coverage	Avg depth	Avg *IC_corpus_*	Coverage + depth gain wrt LCA	Coverage + IC gain wrt LCA
*C. elegans*	*IC_sanchez_*	84.25%	5.29	9.29	**2.76%**	**1.92%**
	*IC_annot_*	80.43%	**5.41**	**9.65**	0.62%	1.41%
	*LCA*	**85.07%**	5.1	9.03	-	-
*D. melanogaster*	*IC_sanchez_*	84.15%	6.9	10.71	**6.31%**	**−0.76%**
	*IC_annot_*	81.11%	**7**	**10.83**	4.42%	−3.08%
	*LCA*	**87.42%**	6.27	10.4	-	-
*D. rerio*	*IC_sanchez_*	84.48%	5.63	9.32	**2.52%**	**0.09%**
	*IC_annot_*	75.51%	**5.95**	**9.53**	−2.02%	−8.08%
	*LCA*	**85.81%**	5.41	9.17	-	-
*R. norvegicus*	*IC_sanchez_*	70.64%	6.04	10.8	**3.71%**	**2.09%**
	*IC_annot_*	66.49%	**6.19**	**11.01**	0.49%	−1.73%
	*LCA*	**71.33%**	5.77	10.48	-	-
*M. musculus*	*IC_sanchez_*	83.22%	6.32	9.32	**6.03%**	**−0.77%**
	*IC_annot_*	72.66%	**6.76**	**9.89**	1.16%	−6.98%
	*LCA*	**85.08%**	5.84	9.19	-	-
*S. cerevisiae*	*IC_sanchez_*	82.48%	6.5	10.9	**3.00%**	**1.74%**
	*IC_annot_*	79.14%	**6.64**	**11.07**	1.20%	−0.69%
	*LCA*	**82.90%**	6.28	10.66	-	-
*H. sapiens*	*IC_sanchez_*	75.56%	6.06	10.74	**3.39%**	**1.65%**
	*IC_annot_*	70.84%	**6.22**	**10.94**	−0.06%	−2.65%
	*LCA*	**76.12%**	5.82	10.49	-	-

For every term in the ontologies, *IC_corpus_* was calculated by considering the main terms and their synonyms, excluding those with less than three characters to avoid too many false positives because of postal codes and other common abbreviations. Note that the results are based on only summaries that are trimmed. The best results among the three algorithms are in boldface.

**Table 4:** Total number of gene summaries for protein coding genes in the Alliance database, for each species and data category

Species	# genes	# genes with summary	Gene MF^a^	Gene BP^b^	Study of gene in human disease	Human ortholog implication in disease	Sub cellular localization	Human ortholog(s)	Expression
*H. sapiens*	*19 348*	*18 483*	*15 317*	*16 801*	*4685*	*NA*	*17 900*	*NA*	*0*
*R. norvegicus*	*23 421*	*20 500*	*16 067*	*17 285*	*1734*	*4465*	*17 855*	*18 757*	*0*
*M. musculus*	*22 982*	*20 488*	*15 855*	*16 858*	*1816*	*4465*	*18 291*	*18 918*	*13 434*
*D. rerio*	*35 496*	*23 571*	*16 426*	*16 326*	*297*	*5363*	*16 378*	*18 475*	*8822*
*D. melanogaster*	*13 999*	*11 980*	*8583*	*9514*	*421*	*2893*	*8862*	*8214*	*9260*
*C. elegans*	*20 124*	*14 730*	*9010*	*9187*	*236*	*2663*	*11 728*	*7286*	*5406*
*S. cerevisiae*	*6604*	*5616*	*4430*	*5130*	*207*	*1170*	*5445*	*3250*	*NA*

NA values indicate that the data category does not apply for that species, whereas zeros indicate that the data are not available at the Alliance.

^a^Molecular function.

^b^Biological process.

As expected, the algorithm based on LCA provides the highest coverage (Table 3, column 3) because common ancestors higher than the LCA do not increase coverage (based on the definition of LCA) and have lower IC values.

Compared with the LCA-based trimming algorithm, IC-based algorithms are able to do the following for the initial set of terms: (i) select one or more common ancestors below the LCA term(s) when they provide a better trade-off between IC and coverage than the LCA and (ii) choose the best subset of initial terms based on IC values when there are no common ancestor terms. Therefore, IC-based algorithms provide similar or lower coverage than the LCA-based one but can provide higher specificity (measured in terms of average depth or average *IC_corpus_* values).

In order to assess whether the gain in terms of specificity outweighs the loss in terms of coverage, we also calculated the average gain in terms of coverage + depth, and coverage + *IC_corpus_*, respectively reported in columns 6 and 7 in Table 3. Both of these measures were calculated by taking the results of the LCA-based algorithm as reference and by subtracting the percentage loss in terms of coverage from the percentage gain in terms of depth and IC. Based on these measures, Table 3 shows that IC-based algorithms outmatch the LCA-based one. This tells us that the loss in terms of coverage of *IC_sanchez_* is marginal compared with the gain in terms of specificity, and although *IC_annot_* always provides the highest specificity in the final summaries, the loss it introduces in terms of coverage with respect to LCA is higher than that introduced by *IC_sanchez_*.

The average depth of the terms selected for inclusion in the summaries by the different trimming algorithms (column 4 in Table 3) indicates that LCA yields an average term depth of 5.78, whereas *IC_sanchez_* results in terms with an average depth of 6.11. This represents, on average, the right term depth in the ontologies to obtain informative and comprehensive gene summaries.

**Figure 4:**
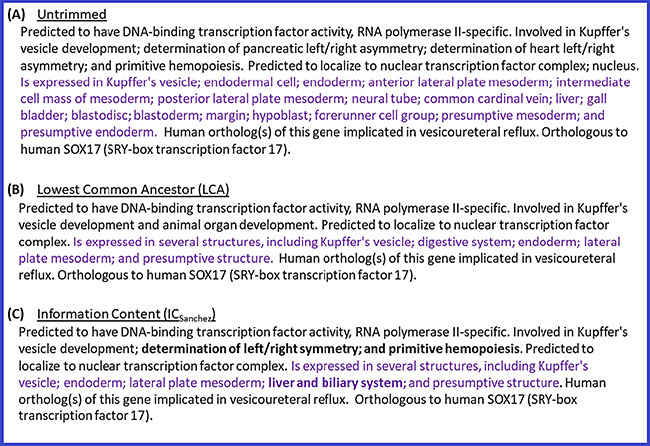
Untrimmed and trimmed summaries for the zebrafish gene *sox17*. (**A**) Untrimmed summary that shows all the 25 terms annotated to the gene. (**B**) The summary trimmed with the LCA-based algorithm. (**C**) The summary trimmed with the algorithm based on IC_Sanchez_. Text highlighted in purple indicates the tissue expression data category, which has 17 terms in the untrimmed summary. Text in bold shows the difference between (B) and (C).

In addition to the above quantitative analysis of the summaries generated with different trimming algorithms, we also performed a qualitative analysis by conducting a manual review of the summaries. This review also confirmed that *IC_sanchez_* yielded the most informative gene summaries. Thus, we decided to use *IC_sanchez_* as the default algorithm for generating gene summaries at the Alliance. Figure 4 shows the summary for zebrafish gene *sox17* with all of the terms for the different data categories (untrimmed), and the trimmed summaries using LCA and IC_Sanchez_ algorithms.

To the best of our knowledge, this is the first paper that shows a comprehensive comparison of different algorithms for gene summary generation and that provides quantitative measures of the quality of a summary, establishing a baseline for future work in the field.

### Numbers and types of summaries

For the 2.3 release version of the Alliance (12 November 2019) a total of 121, 059 gene summaries have been generated for seven species. See Table 4 for total number of summaries for protein coding genes by species and by data category.

## Discussion

### Advantages of the automated gene summaries

Though a fully manually written gene summary for every gene would be ideal, writing and updating of gene summaries is unsustainable because it does not scale. Our solution to this problem has resulted in automated, modular and standardized gene summaries for seven species that are updated with each new release of the Alliance, displayed on Alliance gene pages and available for download. Our method takes advantage of curated data that already exist in the Alliance to generate readable summaries of gene function. Further, it has provided gene summaries for MOKs that lacked them (ZFIN) and supplemented existing summaries at other MOKs. Using consistent vocabulary across all gene summaries may help naive users with quick cross-species gene function comparisons, even though specialized tools may provide better results (38). The application of our method to seven different species demonstrates that our software scales and can be adapted to additional species.

Research in model organisms contributes significantly to the understanding of the pathogenesis of human disease and its alleviation (39). The disease relevance portion of the gene summaries highlights existing animal models of disease (from the six animal species in the Alliance) when available, and/or implications in disease of human orthologs of the MOK gene of interest. This allows human geneticists and biomedical researchers to discover potential new models of disease, without the need to be familiar with specialized disease vocabularies.

### The use of IC measures in other studies

The comprehensive analysis of the different IC measures provided in this paper shows that weighting ontology terms by their IC values during trimming and choosing the right IC measure improved the gene summaries by balancing readability and specificity. Although the IC measure based on the frequency of annotations (*IC_Annot_*, see section ‘Information Content Measures’) resulted in summaries less desirable than those obtained with the measure based on the structure of the ontology (*IC_Sanchez_*), other studies preferred the former measure (40). This means that our results may be specific to the context of gene summaries and may not apply to other contexts.

### Future improvements

Our method for generating gene summaries considers all the terms annotated to a gene; therefore, the key function(s) of a gene may be obscured when there are numerous annotations. The inclusion of other types of data, such as pathway data, would shed more light on key gene function (the Alliance is planning to include such data in the future). Other strategies to improve the current summaries include utilizing the full richness of GO annotations. Currently, the gene summaries algorithm takes into account the GO annotation qualifiers, ‘contributes_to’ and ‘colocalizes with’, when present (http://geneontology.org/docs/go-annotations/#annotation-qualifiers) and generates the appropriate sentence, for example ‘atgp-2 contributes to L-amino acid transmembrane transporter activity’. However, we do not, as yet, use the interontology links (41) between the GO BP and MF ontologies or the forthcoming expanded set of gene product-to-term relations. Use of the BP-MF links would potentially reduce redundancy between the BP and MF summaries and thus improve coverage of the initial set of terms annotated to a gene. Inclusion of gene product-to-term relations such as ‘part of’ or ‘located in’ would result in more accurate gene summaries, for example, that describe a gene product as ‘part of’ a protein complex, or ‘located in’ an organelle, respectively. Further, the use of GO annotation extensions (31) that include information such as the substrate of an enzymatic activity or the cellular location where that activity occurs would provide specific context for gene function.

More recently, the GO consortium has moved to ‘Causal activity modeling (GO-CAM)’, a more complete way of representing the biological function of a gene by connecting the individual GO annotations in order to describe networks or pathways (42). The use of such models would result in richer gene summaries.

Engaging community experts might be another strategy to improve existing summaries. The automated summary could be used as a ‘draft’ that the community expert can edit. Having such drafts makes it easier to add information to a summary, thus lowering the barrier for engagement and possibly increasing participation.

In conclusion, the work presented in this paper represents an improvement over existing gene summary algorithms and has a major practical application as it resulted in thousands of gene summaries at the Alliance, which provide a succinct readable introduction to a gene of interest.
